# The Influence of Emotional Intelligence on Resilience, Test Anxiety, Academic Stress and the Mediterranean Diet. A Study with University Students

**DOI:** 10.3390/ijerph17062071

**Published:** 2020-03-20

**Authors:** Rubén Trigueros, Ana M. Padilla, José M. Aguilar-Parra, Patricia Rocamora, María J. Morales-Gázquez, Remedios López-Liria

**Affiliations:** 1 Department of Language and Education, University of Antonio de Nebrija, 28015 Madrid, Spain; 2 Research Center Hablame, 04005 Almeria, Spain; anapadilla@centrohablame.com; 3 Health Research Centre, Department of Psychology, Hum-878 Research Team, University of Almería, 04120 Almería, Spain; 4 Health Research Centre, Department of Nursing, Physiotherapy and Medicine, University of Almería, 04120 Almería, Spain; rll040@ual.es; 5 Department of Psychology, University of Las Palmas de Gran Canaria, 35001 Las Palmas de Gran Canaria, Spain; mariajose.morales@ulpgc.es

**Keywords:** emotional intelligence, resilience, academic stress, test anxiety, mediterranean diet

## Abstract

The academic transition to university is a turning point in young people’s lifestyles. However, studies to date have focused on student behaviour within the classroom context, rather than on the consequences it may have on their lifestyle. This study aims to analyze the influence of emotional intelligence of university students on their resilience, academic stress, exam anxiety, and eating habits related to the Mediterranean diet at the university stage. This study was carried out with the participation of 733 male and 614 female students from the University of Almeria, aged between 19 and 27. A structural equation model was made to explain the causal relationships between the variables. The results showed emotional intelligence positively predicted resilience. In turn, test anxiety and academic stress were negatively predicted by resilience. Finally, test anxiety and academic stress were negatively predicted by the Mediterranean diet. In short, the results of the present study have shown that academic transfer to university and grading pressure can generate maladaptive consequences for food consumption.

## 1. Introduction

University studies are often experienced by students as a time of great change on a personal level, representing the peak of academic stress due to high workloads, but also because they coincide with a stage of life in which the student must face many changes [1]. Specifically, it coincides with the process of separation from the family, incorporation into the labor market, adaptation to new teachers and colleagues, learning new content that is constantly being updated, curricular reorganizations, and demanding and selective assessments [2]. This situation causes a change in the habits of young people related to the practice of physical activity and food, which can produce a decrease and even abandonment of those behaviors related to healthy habits. However, there are a series of internal mechanisms related to adaptive habits that are essential to overcome the possible vicissitudes that may occur, such as the recognition of emotions and resilience [3,4,5]. Therefore, this study aims to analyze the influence of emotional intelligence on resilience, exam anxiety, academic stress, and academic performance of university students.

### 1.1. Emotional Intelligence

Emotional Intelligence (EI) is a relatively recent term, being understood as the ability to perceive, value, and express emotions accurately; to access and/or generate feelings that facilitate thinking; to understand emotions and emotional knowledge; and to regulate emotions by promoting emotional and intellectual growth [6]. Thus, Mayer, Salovey, Salovey, and Sluyter [7] assume that the components of emotional intelligence are (a) the adequate perception of emotional states; (b) the understanding of their nature; (c) the regulation of them; and (d) all of this in one’s own emotions as well as in those of others.

Recent studies in the field of social psychology have shown a positive relationship between high scores in emotional intelligence and greater psychological well-being, stating that people with greater emotional and behavioral self-control perceive that they have greater control over the demands of their environment and also greater self-esteem. From an educational perspective, emotional intelligence has been positively associated with greater psychological well-being in secondary school students [8], as well as self-efficacy and empathy [9], emotional well-being [10], and academic performance [11]. On the other hand, emotional intelligence has been negatively linked to stress [8], depression [12], and negative emotions [13], elements that lead to the generation of maladaptive behaviors [14]. Even when taking into account the previous studies, studies that relate emotional intelligence and resilience are still quite scarce despite the fact that both factors are related to the successful adaptation of the individual to environmental circumstances [15]. For this reason, negative mood, deregulation of emotions, alterations in routines, and lack of self-esteem are implicated in the multifactorial ethiology of the abandonment of the healthy diet or even eating disorders [16]. The association between mood and eating attitudes has rarely been investigated in university populations. One study with college students found that depressed mood was a predictor of unhealthy eating attitudes. Thus, several studies have shown that increased emotional intelligence leads to more positive attitudes, higher self-esteem, an orientation towards positive values, and greater adaptability [17]; conversely, low levels of emotional intelligence in subjects lead to disordered eating attitudes [18].

### 1.2. Resilence

Resilience is one of the main psychological factors linked to an individual’s adaptation to adverse circumstances. This requires the use of positive reinforcement strategies that help personal well-being. Resilience plays an important role in the academic field as promoting it leads to the development of social, academic, and personal skills, allowing the student to overcome adverse situations [19].

The meta-theory of the resilience affirms the existence of three stages [20]. The first is characterized by the presence of a situation where the individual in physical and mental homeostasis lacks resources or skills to cope with it. In the second stage, the individual tries to readjust to regain the lost balance. Finally, the individual regains balance by acquiring new skills and learning the process. Nevertheless, the meta-theory has a series of limitations that must be taken into consideration. The main limitation is the linearity of the model, that is, in a single event it is the one that opposes the individual [21]. In addition, it does not take into account the protective role that emotions play in the behavior of individuals [22] and as facilitators when dealing with the problem [23]. Fletcher and Sarkar [24] reconceptualized the definition of resilience due to the limitations presented by the meta-theory. They proposed it as a multidimensional psychological capacity that facilitates positive adaptation to danger, thanks to the possession and presence of protective and vulnerable factors outside and inside the individual.

Resilience studies in the educational field have been explored from multiple perspectives, (e.g., [25]) focusing mainly on the external aspects of students and not on what happens during classes or the influence it exerts on adaptive behavior patterns. Despite this, resilience has been found to be positively linked to motivation [26], performance [27], and positive emotions [28]. However, despite the existing studies in the educational field, research is still scarce, focused mainly on the external aspects of students, and not on what happens during classes and the influence it exerts in an academic environment, with respect to other negative psychological variables present in students.

### 1.3. Test Anxiety and Academic Stress

On some occasions, students present a series of emotional reactions that can trigger disinhibition in the face of an exam, damaging their performance during the exam [29]. This negative emotional reaction is understood as anxiety, which is an unpleasant emotional reaction produced by an external stimulus, and is considered by the individual as threatening and thus producing physiological and behavioral changes in the subject before the exams [30]. Tuma and Maser [31] define anxiety as a state that is characterized by the presence of feelings of apprehension, where uncertainty and tension arises as a result of the subject anticipating a real or imaginary threat. We must also distinguish between normal anxiety, which we all have when faced with any major situation (being more physically and mentally active and more prepared to respond), and anxiety that appears continuously and excessively (making behaviors and thoughts uncontrollable) [32].

In this way, exam anxiety can be considered a personality trait or a state. Some people are more prone to exam anxiety, therefore exam anxiety is conceptualized as a specific personality trait in that situation [33]. In contrast, other people perceive the exam situation as potentially threatening due to the fact that failing the exam can interfere with achieving important goals. This generates a state of anxiety that disappears once the exam is passed, and is only present in those exams that the subject perceives as very difficult [34].

Various studies in the academic field have shown that exam anxiety is negatively related to academic performance [35], psychological well-being [36], social relations [37], and physical well-being [38]. In contrast, exam anxiety has been positively related to stress [39], frustration [40], dropping out of school [41], and depression [40].

One of the main triggers of exam anxiety is family and social pressure, in addition to personal pressure focused on obtaining a high academic performance that allows a better future outlook [42]. This continuous pressure can cause the exhaustion of the individual’s reserves and translates into a series of problems, seeing the student unable to react and adapt to the demands of the university environment. When this happens, academic stress appears. In this sense, academic stress is not something proper of the university environment but is present in each one of the stages (e.g., infantile, primary, and secondary), although this stress reaches its zenith during the university period [43].

Different studies in the educational field have shown that stress has been positively associated with depression [44], chronic diseases [45], heart disease [46], with dropping school [47], and in poor academic performance [48].

### 1.4. Objectives and Hypothesis

Considering the aforementioned, this study aimed to analyze the influence of emotional intelligence of university students on their resilience, academic stress, exam anxiety, and eating habits related to the Mediterranean diet at the university stage. The hypotheses are: (a) emotional intelligence will positively predict resilience; (b) resilience will negatively predict exam anxiety and academic stress; (c) anxiety will negatively predict the Mediterranean diet adherence; (d) academic stress will negatively predict the Mediterranean diet adherence.

## 2. Method

### 2.1. Participants

**Figure 1:**
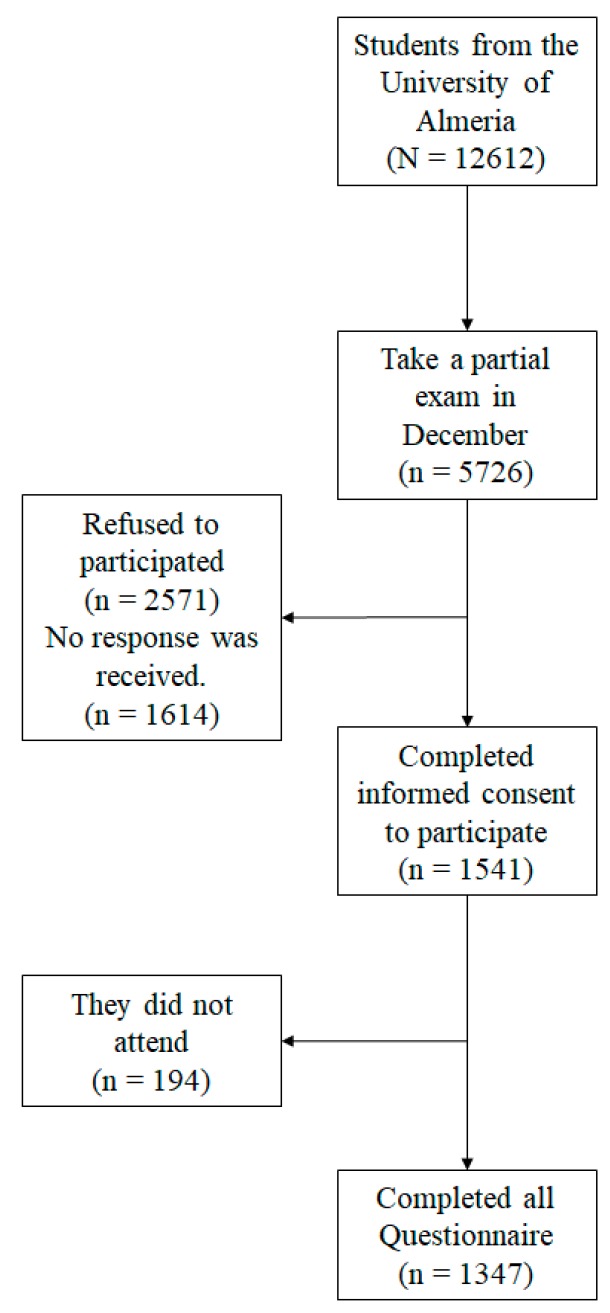
Sample flowchart. N: total; n: subtotal.

The present study was carried out with the participation of students belonging to the University of Almeria (Figure 1), 733 male and 614 female students aged between 19 and 27 (M = 23.58; SD = 2.85).

### 2.2. Measures

#### 2.2.1. Emotional Intelligence

The Trait Meta Mood Scale 24 (TMMS-24) by Fernández-Berrocal, Extremera, and Ramos [49] was used. The scale is made up of 24 items, distributed equally among three factors: emotional attention (e.g., I usually worry a lot about how I feel), emotional clarity (e.g., I almost always know how I feel), and emotional repair (e.g., I try to think positive thoughts even if I feel bad). The students had to assess the degree to which they agreed with each of the items on a 5-point Likert type scale that varied from 1 (very much in agreement) to 5 (very much in disagreement).

#### 2.2.2. Resilience

The Connor-Davidson Resilience Scale [50] was used, validated, and adapted in the Spanish context by Notario-Pacheco, Solera-Martínez, Serrano-Parra, Bartolomé-Gutiérrez, García-Campayo, and Martínez-Vizcaíno [51]. This scale is made up of 10 items (e.g., fate or God helps me) that measure a single factor called resilience. Students had to assess the degree to which they agreed with each of the items on a 5-point Likert scale that varied from 0 (never) to 4 (always). The Spanish version of this scale has been previously used to evaluate university students, presenting adequate reliability [52].

#### 2.2.3. Exam anxiety

The Test Anxiety Inventory [34] was used, validated, and adapted to the Spanish context by Sesé, Palmer, and Pérez-Pareja [53]. The scale is made up of 30 items, distributed among 4 factors: emotionality (8 items; e.g., I feel anxious), worry (10 items; e.g., I’m worried about my exam score), interference (6 items; e.g., I think about anything and get distracted), and lack of confidence (6 items; e.g., I have confidence in my capacity). The students had to assess the degree to which they agreed with each of the items on a 5-point Likert type scale that varied from 1 (almost never) to 4 (almost always).

#### 2.2.4. Academic stress

We used the Student Stress Inventory Stress Manifestations [54], validated and adapted to the Spanish context by Escobar, Blanca, Fernández-Baena, and Trianes [55]. This scale is made up of 22 items, distributed among three factors: emotional (10 items; e.g., I feel worried), physiological (6 items; e.g., I feel a cold sweat), and behavioral (6 items; e.g., I answer the teachers wrong). The students had to assess the degree to which they agreed with each of the items on a 5-point Likert type scale that varied from 1 (not at all) to 5 (totally agree).

#### 2.2.5. Mediterranean diet

The kidmed scale [56] was used, which measures eating patterns related to the Mediterranean diet. This scale is made up of 16 items (e.g., eat a cereal or derivative for breakfast), whose index ranges from 0 to 12. The questions with a negative connotation regarding the Mediterranean diet were evaluated with −1 and those with a positive connotation had a value of +1.

### 2.3. Procedure

**Figure 2:**
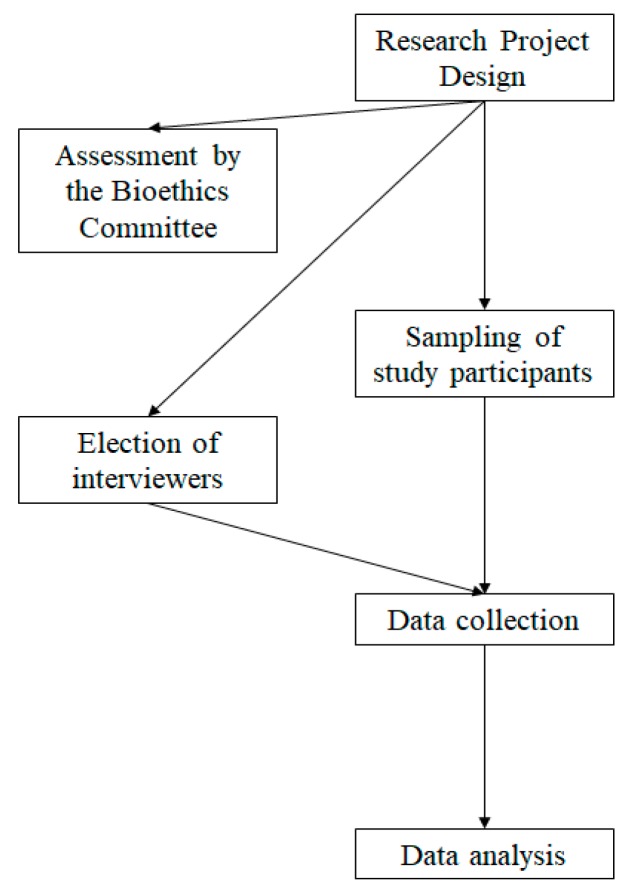
Graphical scheme of design of study.

Figure 2 shows a schematic summary of the study design. Initially, the selection of the participants was non-probabilistically incidental. Furthermore, the criteria for participation in the study were informed consent, voluntary participation and having to take a midterm exam in December.

Later, the permission was sought from various teachers in order to gain access to students and explain the objectives of the study. Later, they were asked to participate voluntarily, giving us their informed consent. The questionnaires were completed in the third week of December 2019, immediately prior to the start of the mid-term examination period. The students completed the questionnaires anonymously and respected all ethical procedures, with a member of the research group present to resolve any doubts that arose. The estimated time to complete the questionnaire was around 20 min.

The study was approved by the bioethics committee of the University of Almeria (Ref. UALBIO 2019/014) and respected all the procedures established by the Declaration of Helsinki.

### 2.4. Data Analysis

The statistical program SPSS version 25 (IBM, Armonk, NY, USA) was used to perform the descriptive statistical analyses, reliability analyses and bivariate correlations. In addition, AMOS version 20 (IBM, Armonk, NY, USA) statistical program was used to perform the structural equation model (SEM) to analyze the relationships established in the hypothesized model.

**Figure 3:**
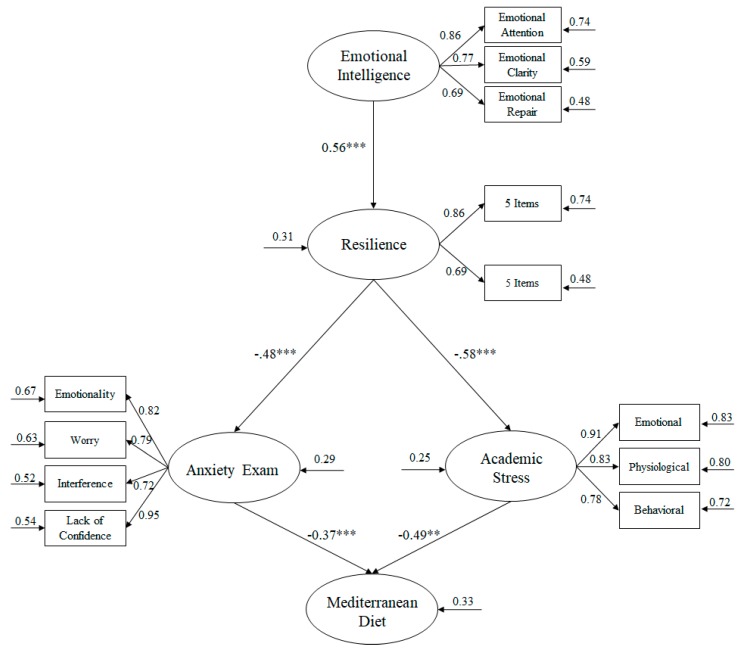
Relationship between variables through a structural equation model (SEM). All the relationships are significant, showing the variance on the small arrows. Note: *** *p* < 0.001; ** *p* < 0.01.

The bootstrapping procedure was used together with the maximum likelihood method to analyze the hypothesized model (see Figure 3). The estimators were not affected by the lack of normality and were therefore considered robust [57]. The following adjustment rates were taken into account in order to accept or reject the hypothesized model [58]: Incremental rates (IFI, Incremental Fit Index; CFI, Comparative Fit Index; and TLI, Tucker Lewis Index) must show a score above 0.95; the error rates Root Mean Square error of Approximation (RMSEA) and Standardized Root Mean Square Residual (SRMSR), are considered acceptable values equal to or less than 0.06 and 0.08 respectively; and finally a value *χ^2^/df*, being considered acceptable values lower than 3.

## 3. Results

### 3.1. Preliminary Analysis

The mean, standard deviation, and bivariate correlations are shown in Table 1. Correlation analyses showed a positive correlation between emotional intelligence, resilience, and the Mediterranean diet, while a negative correlation was found for academic stress and exam anxiety. On the other hand, academic stress and exam anxiety reflected a positive correlation. In addition, Table 1 shows the reliability analyses through Cronbach’s α for each of the factors, reflecting scores above 0.70 [59].

### 3.2. Structural Equation Model Analysis

In view of the complexity of the hypothesized model, the number of latent variables was reduced by at least two indicators [60]. In this way, a SEM was carried out to analyze the relationships between each of the study’s variables. The latent variables used were: emotional intelligence included three indicators [49]; exam anxiety included four indicators [53]; academic stress [55]; and finally, resilience was necessary to divide the 10 items of the scale into two indicators, as suggested by McDonald and Ho [60].

The hypothesized predictive relationship model (Figure 3) showed that the adjustment rates were adequate: *χ*^2^ (61, *N* = 1347) = 186.34, *χ*^2^/*df* = 3.05, *p* < 0.001, IFI = 0.96, TLI = 0.96, CFI = 0.96, RMSEA = 0.049 (CI 90% = 0.045–0.053), SRMSR = 0.039. These results were in line with the established parameters, therefore the proposed model was accepted as adequate. Similarly, the contribution of each of the factors to the prediction of other variables was examined through standardized regression weights.

Following this, the relationships obtained between the different factors integrated in the model were described (Figure 3):

(a) Emotional Intelligence positively predicted resilience (β = 0.56, *p* < 0.001).
(b) Resilience negatively predicted anxiety exam (β = −0.48, *p* < 0.001) and academic stress (β = −0.58, *p* < 0.01).
(c) Anxiety exam negatively predicted Mediterranean diet (β = −0.37, *p* < 0.001).
(d) Academic stress negatively predicted Mediterranean diet (β = −0.49, *p* < 0.01).

## 4. Discussion

The aim of this study was to analyze how emotional intelligence influences resilience, academic stress, exam anxiety, and the balanced diet represented by the Mediterranean diet in university students. This study does not focus mainly on the motivational processes and/or the academic performance of university students, as is the case in the various studies to date. This study focuses mainly on emotional intelligence and resilience of students. In this sense, if university students are capable of overcoming adversity and recognizing their own and other people’s emotions, controlling them and projecting them in an appropriate way can give rise to a series of adaptive behaviors that can favor the adoption of healthy life habits and higher academic performance [61].

The results of this study show how emotional intelligence positively predicted resilience. However, these results are not comparable to similar studies in the university context, although they are in similar contexts such as teaching. In this sense, a study carried out by Trapp [62] with secondary school education teachers, showed that those who possessed high levels of emotional intelligence had high levels of resilience. Similarly, a study conducted with secondary school students showed that those students who possessed high levels of emotional repair and clarity were associated with high levels of resilience [63]. Thus, the results of the present study are similar to the results shown in previous studies, as emotional intelligence can contribute to the satisfactory adaptation to the different contingencies of life and to the development of a set of meta-qualities that can be practiced, learned, and applied to the capacity for recovery [15]. Therefore, both factors can be considered as a characteristics or features of the individual’s personality, which can help him/her to successfully adapt to all the pressures to which students are subjected during that stage.

In addition, the results have shown that resilience negatively predicted exam anxiety and academic stress. These results are been similar to several studies in the field of clinical psychology, where it has been found that resilience has a protective effect on anxiety and depression in adolescents (e.g., Hjemdal, Vogel, Solem, Hagen, and Stiles, [64]). Similarly, a study by Bonanno, Kennedy, Galatzer-Levy, Lude, and Elfström [65] in patients who have suffered a spinal cord injury showed that those with high levels of resilience were less likely to suffer from anxiety or depression. Furthermore, the results of the present study have shown resilience negatively predicted academic stress. These results are similar to studies in the field of medicine, where research conducted by Fang et al., [66] with human immunodeficiency virus infected patients with high levels of resilience were more likely to cope with the disease, have lower levels of stress and a higher quality of life. Similarly, in a study conducted by Dumont and Provost [67] from the field of social psychology with adolescents, it was observed that those who had high levels of resilience showed social activity and less chance of suffering stress and/or depression. However, as with the relationship between resilience and exam anxiety, we have no evidence from previous studies relating resilience to academic stress in the university setting. Thus, the present study shows the importance of promoting the adaptability of university students to the multiple challenges they have to face in this new academic stage. To this end, it is essential to introduce courses and activities aimed at promoting intrapersonal and interpersonal skills, competence, optimism, self-concept, and autonomy into educational programs [19,68].

Finally, the results of the study have shown exam anxiety and academic stress negatively predicted the Mediterranean diet. However, we have very little evidence from studies that have analyzed this relationship in the university academic environment, or in other areas such as medicine or clinical and social psychology. In this sense, studies related to anxiety and eating habits with young people and adolescents between 8 and 18 years, such as the study conducted by Bektaş, Uğur, Gençtürk, Aysev, Sireli and Deda [69], have shown that those who had high levels of anxiety manifested maladaptive behaviors such as sedentary, unbalanced diet, or even eating disorders. Similarly, a study by Pastore, Fisher, and Friedman [70] of high school students showed that those with high levels of self-esteem had balanced eating habits and a lower risk of eating disorder-related illnesses compared to high school students with high levels of anxiety. On the other hand, studies that have explored the relationship between stress and eating in children, such as the research conducted by Kim and Lee [71], have shown that high levels of stress were a predictor of high consumption of foods that were also related to high caloric value. Similarly, a study conducted in the adult population by Kim and Kim [72] showed that those with high levels of stress were related to poor or excessive eating and could even be the cause of various eating disorders, such as bulimia or anorexia. Thus, the results of this work suggest that the academic environment can also have maladaptive effects contrary to personal progress and quality of life. Therefore it is essential to have psychological intervention programs aimed at prevention and education of young university students, where many of them live the experience of emancipation for the first time. These circumstances may require them to make constant adjustments in their day-to-day life, which together with academic stress and the pressure of exam grades can cause them to abandon the balanced diet typical of the Mediterranean diet [73].

Despite the results obtained in this study, a number of limitations should be noted. First, the study is largely based on self-reported questionnaires. Second, the selection of the participants was non-probabilistically incidental. Third, this was a relational study, which did not permit causal relationships to be identified. In this way, the results can be interpreted according to each individual’s perspective. Future studies should analyze the influence of the social context on the development of emotional intelligence and resilience, in addition to analyzing the incidence on academic stress and exam anxiety.

## 5. Conclusions

The present study analyses for the first time the influence of emotional intelligence on resilience, test anxiety, academic stress, and adherence to the Mediterranean diet.

The results obtained in the present study show the positive association of emotional intelligence with respect to resilience, and how this was negatively related to test anxiety and academic stress. Finally, test anxiety and academic stress were negatively related to the Mediterranean diet.

## Figures and Tables

**Table 1:** Descriptive statistics and correlations between all variables.

Factors	*M*	*SD*	α	1	2	3	4	5
1. Emotional Intelligence	4.11	0.68	0.81	-	0.66 ***	−0.45 ***	−0.37 ***	0.23 *
2. Resilience	1.36	1.00	0.86		-	−0.49 **	−0.71 **	0.35 **
3. Exam Anxiety	5.82	1.08	0.83			-	0.53 ***	−0.54 ***
4. Academic Stress	4.67	1.43	0.85				-	−0.61 ***
5. Mediterranean Diet	5.23	1.97	-					-

Note: * *p* < 0.05, ** *p* < 0.01; *** *p* < 0.001
